# Molecular differentiation between complete and incomplete responders to neoadjuvant therapy in rectal cancer

**DOI:** 10.21203/rs.3.rs-4456000/v1

**Published:** 2024-07-01

**Authors:** Zechen Chong, Fengyuan Huang, M. McLeod, Regina Irwin, Mary Smithson, Zongliang Yue, Min Gao, Karin Hardiman

**Affiliations:** University of Alabama at Birmingham; University of Alabama at Birmingham; University of Alabama at Birmingham; University of Alabama at Birmingham; University of Alabama at Birmingham; Auburn University; University of Alabama at Birmingham; University of Alabama at Birmingham

**Keywords:** neoadjuvant therapy, rectal cancer, genomic and transcriptomic characteristics

## Abstract

**Background:**

Neoadjuvant chemoradiotherapy (nCRT) is the standard treatment for locally advanced rectal cancer, but only 20–40% of patients completely respond to this treatment.

**Methods:**

To define the molecular features that are associated with response to nCRT, we generated and collected genomic and transcriptomic data from 712 cancers prior to treatment from our own data and from publicly available data.

**Results:**

We found that patients with a complete response have decreased risk of both local recurrence and future metastasis. We identified multiple differences in DNA mutations and transcripts between complete and incomplete responders. Complete responder tumors have a higher tumor mutation burden and more significant co-occurring mutations than the incomplete responder tumors. In addition, mutations in DNA repair genes (across multiple mechanisms of repair) were enriched in complete responders and they also had lower expression of these genes indicating that defective DNA repair is associated with complete response to nCRT. Using logistic regression, we identified three significant predictors of complete response: tumor size, mutations within specific network genes, and the existence of three or more specific co-occurrent mutations. In incompletely responder tumors, abnormal cell-cell interaction and increased cancer associated fibroblasts were associated with recurrence. Additionally, gene expression analysis identified a subset of immune hot tumors with worse outcomes and upregulated of immune checkpoint proteins.

**Conclusions:**

Overall, our study provides a comprehensive understanding of the molecular features associated with response to nCRT and the molecular differences in non-responder tumors that later reoccur. This knowledge may provide critical insight for the development of precision therapy for rectal cancer.

## Background

Colorectal cancer is the third leading cause of cancer death in the United States [1]. Rectal cancer constitutes about one-third of cases. Stage 2 and 3 rectal cancers predominantly undergo treatment with neoadjuvant chemoradiation (5FU and 2 Gy radiation for six weeks) and often chemotherapy (FOLFOX), followed by surgical excision using the total mesorectal excision technique [2]. Responses to this neoadjuvant treatment vary substantially [3], with complete response rates between 20–40% [4]. Complete responders have reduced risk of local recurrence and metastasis [5, 6] and as demonstrated in a recent multicenter trial, these patients may not need to undergo surgical excision [4]. The lack of predictors of response remains a significant obstacle to improving treatment outcomes.

Prior research has assessed clinical predictors of complete response, including increased serum carcinoma-embryonic antigen (CEA), imaging-detected extra-mural venous invasion and tumor size, primary tumor and regional lymph nodes staging (T and N stages) [7]. Clinical predictors that predict overall worse outcome generally predict worse response to neoadjuvant therapy, but no prior study has identified genomic predictors that are used clinically to predict which patients will be complete responders to neoadjuvant treatment.

As technology advances, studies have begun to assess alterations in DNA, RNA, and individual proteins as well as immune cells as predictors of response [8–12]. Smaller studies of individual or small groups of genetic alterations have yielded mixed results. A recent meta-analysis that assessed the prognostic role of specific genomic mutations (*RAS, TP53, BRAD, PIK3CA, and SMAD4*) in predicting complete response found that only *KRAS* mutation was significantly associated with incomplete response [13]. Most of these studies had small sample sizes (< 30 patients) and didn’t integrate RNA and DNA sequencing data [3, 14]. The larger dataset from the Cancer Genome Atlas (TCGA) rectal cancer cohort excludes treated tumors and thus, this is unable to address the question of why patients respond differently[15]. A recent study utilizing genomic data on 738 rectal cancer patients provided a summary of genomic data but lacked in-depth integrated analysis assessing for predictors of response [16].

Our study combined genomic sequencing data from pre-treatment biopsies of 20 rectal cancer patients from our group with publicly available data from 692 patients, excluding patients with stage 4 disease and those treated with chemotherapy only [16]. Because our data was collated from multiple studies, it included patients who had a variety of treatments, but all underwent treatment with 5-fluorouracil plus long course radiation as part of their treatment. We performed an in-depth integrated analysis to identify response-associated alterations and features linked to future recurrence. Our findings revealed multiple differences between CR and ICR patients, including distinct mutation networks, DNA repair alternations, tumor mutation burden (TMB), and immune cell infiltrate. We performed logistic regression to identify predictors of complete response and assessed ICR tumors for recurrence-associated features and identified that recurrent tumors had decreased immune cells, increased cancer-associated fibroblasts, and mutations affecting TGF-ß signaling and cell-cell interaction pathways. Overall, we present a novel and comprehensive evaluation of predictors of rectal cancer outcomes after neoadjuvant therapy.

## Methods

### Patients

**Figure 1:**
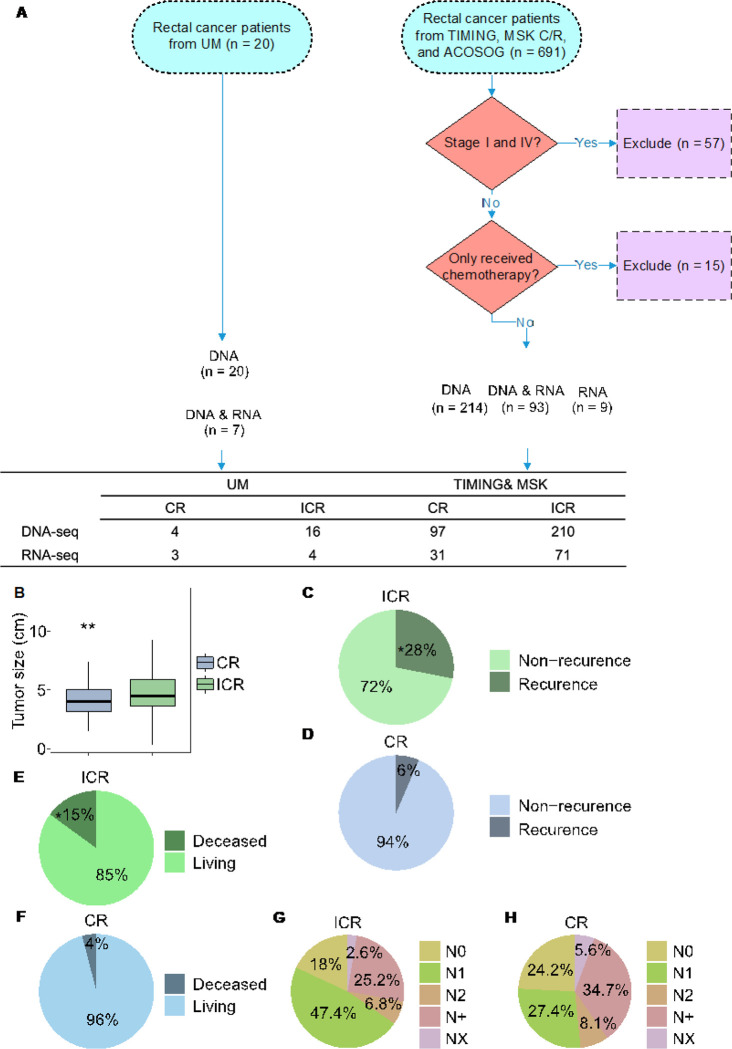
Summary of Data sources and clinical differences in response to neoadjuvant therapy (nCRT). **A:** Overview of the datasets used in the study. UM: University of Michigan cohort; TIMING: Timing of Rectal Cancer Response to Chemoradiation cohort; MSK C +R: Memorial Sloan Kettering Research + Clinical cohort; ACOSOG: ACOSOG Z6041 cohort. Patient selection is depicted, including exclusion criteria and the counts of patients whose DNA and RNA were sequenced. **B:** Tumor Size Distribution by Response to nCRT. Box plots display tumor sizes comparing complete responders (CRs, n=124) to neoadjuvant chemoradiation therapy (nCRT) with incomplete responders (ICRs, n=267). Statistical analysis using the Mann-Whitney U test shows a significant difference (p = 0.0069). Sample size for the analysis totaled 391 patients. **C-H:** Pie charts showing the fractions of recurrent (**C**,**D**), overall survival status (**E, F**), and N stages (**G, H**) in ICR tumors (n=267) and CR tumors (n=124), respectively. An asterisk in panel C signifies a statistically significant difference as determined by the Chi-squared test (p < 0.05).

For this study, we identified and analyzed available data for patients who had pre-treatment tumor biopsies with subsequent DNA and/or RNA sequencing of their tumor, received chemoradiation, had subsequent surgical resection or clinical followup as part of a trial, and had available data regarding tumor response to treatment. Tumors were from four cohorts: University of Michigan (UM) (20 patients), Timing of Rectal Cancer Response to Chemoradiation (TIMING), ACOSOG Z6041, and Memorial Sloan Kettering (MSK) Research and Clinical cohorts (691 patients) were included, totaling 711 patients with confirmed diagnosis of rectal adenocarcinoma (Fig. 1). The UM patients underwent treatment with 5FU and 54Gy radiation over 5.5 weeks (chemoradiation) followed by surgery 8–12 weeks later. The TIMING trial patients got chemoradiation followed by differing numbers of cycles of FOLFOX (5FU, oxaloplatin, and leukovorin) prior to surgical resection. The patients in ACOSOG Z6041 cohort were treated with neoadjuvant chemoradiotherapy consisting of 50 Gy of radiation, capecitabine and oxaliplatin followed by local excision. The MSK cohort got chemoradiation either before or after 8 cycles of FOLFOX and patients who appeared to have a clinical complete response on endoscopy and imaging were followed. For our study, we considered patients to have a CR if they underwent surgical resection and had a CR or if they were a persistent clinical CR in the MSK dataset but did not undergo surgical resection. All genomic and associated clinical data for MSK and TIMING were downloaded from the cBioPortal (https://www.cbioportal.org/study/summary?id=rectal_msk_2022). Raw RNA sequencing data from all cohorts were downloaded from GEO under the accession numbers: GSE242786 and GSE209746.

### Whole Exome Sequencing

For the UM cohort, genomic DNA samples were fragmented to a target size of 300 bp using the Covaris S2 system. The samples were end-repaired, poly A-tailed, and ligated with custom adapters using the NEBNext DNA Library Prep kit. These adapters had 6 bp barcodes designed with BARCRAWL software^48^ and synthesized by Integrated DNA Technologies. After ligation, they were size selected to 300 bp on a 2% agarose gel, retaining 1-mm gel slices. The samples were isolated from the gel using the Qiagen QIAquick system. Ligation products (10 or 15 μl) were enriched using the Phusion master mix kit and underwent 14 PCR cycles. PCR products were purified using AmpureXP beads. Library quality was checked using the Agilent Bioanalyzer and qPCR. The libraries were captured using the Nimblegen SeqCap EZ V3 Exome Enrichment Kit and sequenced on the Illumina HiSeq 2000 with 100 bp paired-end reads using v3 reagents.

### Somatic Mutation Detection

For detecting somatic single nucleotide variant (SNV) and insertions/deletions (INDELs), the best practice guidelines of GATK (v4.0.11) [17] (https://software.broadinstitute.org/gatk/best-practices/workflow?id=11146) were followed, and strelka2 (v2.9.10) [18] was employed. Somatic variants with a depth < 30 and Allele Fraction (AF) < 0.1 were excluded. The remaining mutations were annotated using ensembl-vep (v109.3) [19]. The VCF files were converted to MAF format using vcf2maf (v1.6.21) [20]. Analysis focused on mutations in coding regions. A mutation rate (total mutations/length of coding regions) was calculated for all somatic mutations. A propensity score weighting algorithm (PSW) [21] was applied to balance confounding factors. The propensity score was calculated based on the response type (CR and ICR) using a logistic regression. Samples were re-weighted using the Matching Weight scheme (MW) [22]. This process included balanced checking steps that revised the calculation until standardized differences of all covariates were less than 10%, balancing confounding factors between CR and ICR patients. Next, mutation rates between the two balanced groups were compared using a weighted t-test.

### Significantly Mutated Genes Detection

MutSig2CV [23] was applied to identify significantly mutated genes in tumors with complete response and incomplete response to nCRT. Significantly mutated genes were mutated beyond random expectations, considering the background mutation rate and mutational processes. Genes with *q*-value < 0.05 in CR tumors, but > 0.05 in ICR tumors were defined as CR-specific, and vice versa for ICR-specific genes. Functional enrichment analysis of SMGs was performed using Enrichr [24]. Significantly enriched Gene Ontology (GO) and Kyoto Encyclopedia of Genes and Genomes (KEGG) pathways were identified with a threshold p-value < 0.05.

The mutated genes with > 5% mutation frequency were selected for comparison between CR and ICR, including recurrent and nonrecurrent ICR tumors. Lollipop plots were generated using the maftools (v3.15) [25] package. Co-occurring and mutually exclusive mutation pairs were analyzed using the maftools package. Interactions of top 25 mutated genes were visualized using the ggplot2 package. Networks and interactions of these mutations were explored in the STRING database [26] and illustrated via Cytoscape (v3.10.0) [27].

### RNA Sequencing

From our cohort, RNA was isolated from fresh frozen pre-treatment biopsy samples using the Allprep kit (Quiagen). Total RNAs were used to generate mRNA sequencing libraries and sequenced on the Illumina HiSeq 2000 platform. The TIMING and MSK samples were treated similarly [16].

### Transcriptomic Analyses

Raw FASTQ files were assessed using FastQC (v0.11.9). Single-end RNA-seq reads were aligned to the human genome (GRCh38) with STAR software (v2.6.1) [28]. Gene expression levels were calculated from BAM files using HTSeq (v0.11.2) [29]. Raw read counts were normalized, and genes with a p-value ≤ 0.05 were identified as differential expression genes (DEGs) using the Deseq2 package (v3.14) [30]. Functional enrichment of DEGs was performed through Enrichr [24]. Significant GO terms and KEGG pathways were identified with p-value < 0.05. Furthermore, Gene set enrichment analysis (GSEA) [31] was conducted to determine gene sets that were significantly different between response and non-response groups. The H (h.all.v6.2.symbols.gmt) sub-collection from the Molecular Signatures Database (http://software.broadinstitute.org/gsea/msigdb/index.jsp) served as the reference for GSEA, with a normalized enrichment score (NES) and false discovery rate (FDR) denoting statistical relevance.

For the validation of subset ICR tumors using TCGA rectal tumors (READ), the gene expression data processed using the second TCGA analysis pipeline (RNAseqV2) were collected. Only tumors in stage II and III with consensus molecular subtypes (CMS) subtypes and microsatellite instability (MSI) status, were included. The hierarchical clustering method was used to identify cluster 4-like tumors based on the DEGs between cluster 4 and other clusters.

### Immune Cell Infiltration Analyses

Based on gene expression, abundances of infiltrating immune cells were estimated using TIMER2.0 [32] which includes TIMER, CINERSORTx [33], quanTIseq [34], xCell [35], MCP-counter [36] and EPIC [37] algorithms. Significant differences in immune cells between tumor groups were defined at the cutoff of *p*-value = 0.05 using the Mann Whitney U test.

### Survival Analyses

The “Survival” package (v3.3–1) was used to build a standard survival object. To evaluate gene expression on survival, Kaplan-Meier survival curves were generated. Patients were stratified into high and low expression groups based on mean expression levels. Survival curves for these groups were compared, and the log-rank test was used to evaluate significant differences in survival durations (*p*-value < 0.05).

### Logistic Regression Model for Complete Response

To identify potential predictors of tumor response, a logistic regression model [38] was fit to clinical and genetic data. First, bivariate comparisons using Fischer’s exact test were made between tumor response and binary indicators for gene mutations and co-occurring mutations, with factors at *p*-value < 0.1 combined into variables measuring counts of particular mutated genes and counts of co-occurring mutations. Bivariate comparisons were also made between tumor response and patient demographics (age, gender, race, BMI) and tumor characteristics (size, stage, MSI status). A logistic regression model for complete response was then fit including tumor size, tumor stage, and the variables indicating the number of identified gene mutations of interest and number of co-occurring mutations of interest. Count variables were explored as both continuous and categorical and a final model was selected with the highest R^2^ Tjur and lowest AIC. The final functional form of mutation count variables consisted of a binary indicator for any mutation in a gene of interest and a categorical variable indicating 0, 1 to 2, or 3 or more co-occurring mutations of interest. Model performance metrics including sensitivity, specificity, positive prediction value (PPV), and negative prediction value (NPV) were evaluated on the training data using the R package pROC [39].

### Statistical Analysis

Statistical analyses were conducted using R Studio (v1.2.1335) and R (v3.6.1). Specific R packages and methods used in each step are described in the preceding sections.

## Results

This study analyzed 711 stage 2 and 3 rectal cancer patients who received chemoradiation post-biopsy from four cohorts: 20 from University of Michigan (UM), and 691 from the ACOSOG (n = 25), Timing (n = 68) and Memorial Sloan Kettering (MSK, n = 598) groups. We excluded 57 patients who only received chemotherapy and 15 with stages I or IV tumors. Subsequently, 336 patient tumors with available DNA or RNA sequencing data were used for our genomic analyses (Fig. 1A). Those showing pathological or clinical complete response were categorized as complete responders (CRs), while the rest were classified as incomplete responders (ICRs). Significant clinical differences were identified between CR (n = 124) and ICR groups (n = 267) (**Supplementary Fig. 1**). Notably, ICR patients exhibited larger tumor sizes (Mann Whitney U test, *p*-value < 0.05, Fig. 1B), higher recurrence rates (Chi-square test, *p*-value < 0.05, Fig. 1C–D) and deceased overall survival (Chi-square test, *p*-value < 0.05, Fig. 1E–F). Furthermore, 79.4% of ICR patients were lymph nodes positive (N1, N2, N+), compared to 70.2% of CRs (Fig. 1G–H). These results align with earlier research linking complete response and reduced recurrence [40]. Notably, treatment approach was not significantly associated with the type of response; comparison between CR and ICR showed no statistically significant difference in response to chemoradiation therapy whether it was administered as a consolidation or as the only form of treatment (Chi-square test, p-value = 0.373) (**Supplementary Table 1**). In addition, our analysis indicated that microsatellite instability (MSI) status did not significantly distinguish between CR and ICR outcomes (Chi-squared test, p-value 0.233) (**Supplementary Table 1**).

### Genomic characteristics of response to nCRT

**Figure 2:**
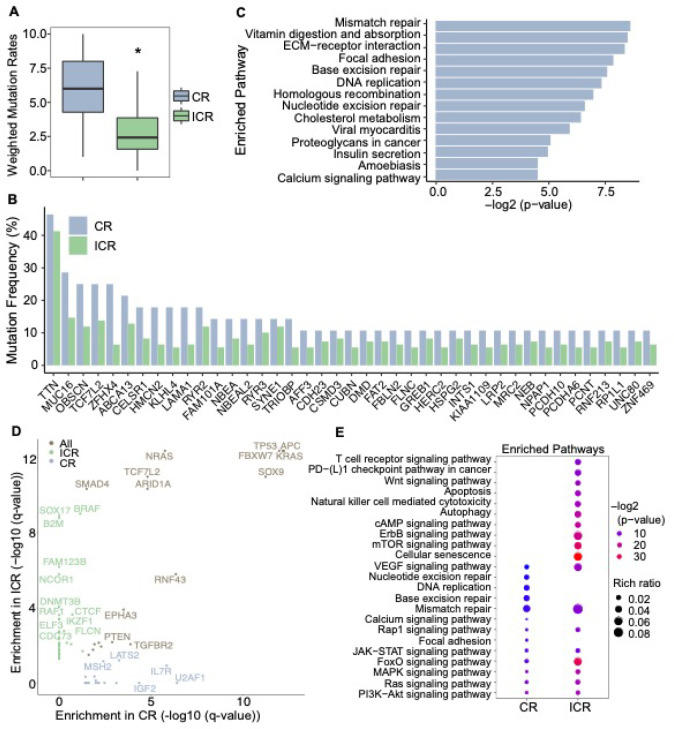
Mutation rates and significantly mutated genes (SMGs) in CR and ICR tumors. **A:** Mutation Burden Comparison. Mutation rates are compared between complete responders (CR, n=28) and incomplete responders (ICR, n=109) using a weighted t-test, showing a significant difference (p=0.0369). **B**: Mutational Frequencies in CR vs. ICR Tumors. Bar plot indicates higher mutation frequencies in CR tumors (n=104; microsatellite instability [MSI]=7; no matched control [NMC]=12) compared to ICR tumors (n=260; MSI=7; NMC=27). **C**: Pathway Enrichment in CR Tumors. Bar plot demonstrates pathways significantly enriched with frequently mutated genes in CR tumors. **D**: Dot plots showing Significantly Mutated Genes (SMGs) enriched in CR tumors (lightsteelblue, n=104), ICR tumors (darkseagreen, n=260), and both groups (wheat), *q*-value < 0.05 after correction for multiple hypothesis testing. **E**: Enriched pathways for SMGs. Pathways significantly enriched for SMGs are shown, with the rich ratio indicating the proportion of SMGs relative to the total number of genes in each pathway.

Prior study has indicated that complete response to nCRT is associated with a high tumor mutation burden (TMB) in rectal cancer [41] but potential confounding factors were not accounted for such as BMI, gender, and age. To do so, we applied the propensity score weighting algorithm in the TMB comparison (**Supplementary Fig. 2A**). Even after this adjustment, CR tumors exhibited significantly higher mutation rates (Fig. 2A). Importantly, this increased mutation rate in CR tumors was not influenced by the presence of microsatellite instability (MSI) in tumors (**Supplementary Fig. 2B**). This finding implies that TMB may be an important factor for predicting the response to neoadjuvant therapy.

In addition to TMB, we also aimed to identify the relevant genetic features associated with response. We first focused on the frequently mutated genes and compared their mutation frequencies between CR and ICR tumors. The commonly mutated colorectal cancer-related genes, such as *TTN, APC, TP53*, and *KRAS* were found in a high percentage of rectal tumors (Fig. 2B, **Supplementary Fig. 2C-D)**. However, genes like *APC, TP53, KRAS, PIK3CA* and *FBXW7* displayed higher mutation frequencies in ICR tumors (**Supplementary Fig. 2D**). Interestingly, genes with higher mutation frequencies in CR tumors were enriched in several DNA repair pathways (Fig. 2C), including Mismatch repair (MMR), Base excision repair (BER), Homologous recombination (HR), and Nucleotide excision repair (NER). This observation suggests a potential association between complete response to nCRT and DNA repair deficiency.

Next, we used MutSig2CV [23] to identify significantly mutated genes (SMGs) for CR and ICR tumors, respectively. MutSig2CV identifies genes that are mutated more frequently than expected by chance, considering three factors: background mutation rate, localized hotspots, and vertebrate conservation. We discovered 36 SMGs in ICR tumors and 17 in CR tumors (Fig. 2D, **Supplementary Table 2**). Several well-known cancer driver genes, including *TP53, APC, KRAS, NRAS, ARID1A*, and *PIK3CA*, were present in both groups. Notably, consistent with the frequently mutated genes in CR tumors (Fig. 2D), the CR-specific SMGs were enriched in DNA repair pathways, while the ICR-specific SMGs were predominantly associated with oncogenic signaling pathways (Fig. 2E).

**Figure 3:**
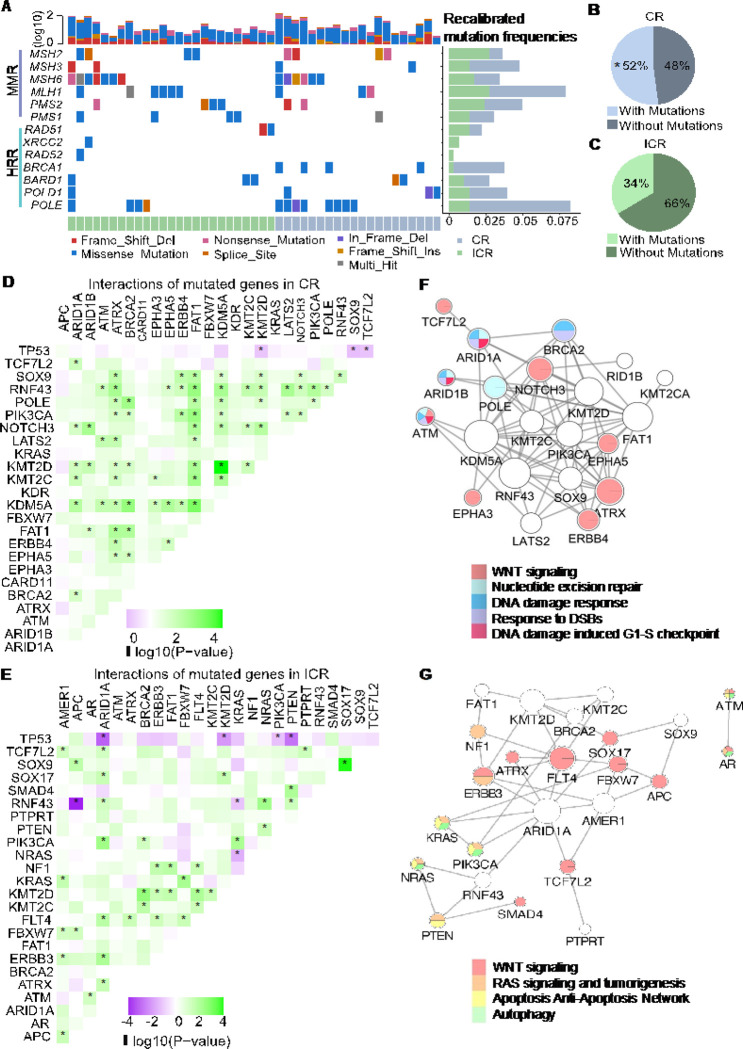
Genomic determinants of response to nCRT. **A:** Oncoplot illustrating mutations in DNA repair-related genes. MMR: DNA mismatch repair. HRR: Homologous recombination repair. The top bar plot quantifies mutation frequency on a log10 scale for each patient. The side bar plot recalibrates mutation frequencies across individual genes, with a sample size of 364 (CR=104, ICR=260). **B**, **C**: Pie charts representing the distribution of patients with and without mutations in DNA repair-related genes for complete responders (CR) (**B**) and incomplete responders (ICR) (**C**). In CR patients, 52% exhibited mutations (* indicates statistical significance, Chi-squared test, p = 0.002), while in ICR patients, 34% had mutations. **D**, **E**: Diagrams depicting interactions between frequently mutated genes in CR tumors (**D**) and ICR tumors (**E**), with green indicating co-occurring mutations and purple showing mutually exclusive mutations. Significant interactions determined by Fisher’s exact test are marked with stars (p<0.05). **F**, **G**: Networks of co-occurring mutated genes in CR tumors (**F**) and ICR tumors (**G**).

Furthermore, we specifically assessed for mutations in genes associated with various mechanisms of DNA repair. After balancing confounding factors, we observed higher mutation frequencies in *MSH3*, *MLH1, PMS1, BRCA1, BARD1, POLD1*, and *POLE* in CR than ICR tumors (Fig. 3A). Overall, a significant higher proportion of CR patients (52% vs. 34%, *p*-value < 0.05) had DNA repair-related mutations compared to ICR (Fig. 3B–3C). These DNA repair alterations were not related to MSI status as they were rarely found in MSI tumors and MSI-H status was very similar and rare between CR and ICR tumors (2/104 CR and 2/260 ICR tumors).

In cancer, certain mutations exhibit evolutionary dependencies [42], reflecting interactions between genes. Alteration in one gene may influence the mutation pattern of another, thereby creating a complex network of mutually exclusive or co-occurring mutation pairs [43]. To explore whether specific genetic interactions are associated with the response to nCRT, we detected co-occurring and mutually exclusive mutation pairs within CR and ICR tumor groups. Interestingly, CR tumors demonstrated more significantly co-occurring mutations in cancer-related genes, with 70 co-occurring pairs in CR tumors compared to 38 pairs in ICR tumors (Fig. 3D and 3E, **Supplementary Data 1**, Fisher’s exact test, *p*-value < 0.05). In CR tumors, genes with co-occurring mutations were primarily involved in DNA damage response, double-strand DNA break (DSB) response, and nucleotide excision repair (NER) (Fig. 3F). In contrast, the gene network with co-occurring mutations in ICR tumors was predominantly associated with tumorigenic pathways, such as RAS signaling, autophagy, apoptosis, and anti-apoptosis (Fig. 3G).

Additionally, we identified 7 significantly mutually exclusive mutation pairs in ICR tumors and 3 in CR tumors (Fisher’s exact test, *p*-value < 0.05) (**Supplementary Data 1**). Alterations in *KMT2D* and *TP53* were observed in both CR and ICR groups. The mutually exclusive alterations in *APC* and *RNF43* has been reported in microsatellite-unstable colorectal adenocarcinomas [44]. Mutations in *PTEN* and *TP53* were also exclusive in a subgroup of colorectal cancer [45] and breast cancer [46]. Similar exclusivity between *TP53* and *ARID1A* has been observed in other cancer types [47].

### Transcriptomic differences and tumor immune cell infiltrate in response to nCRT

**Figure 4:**
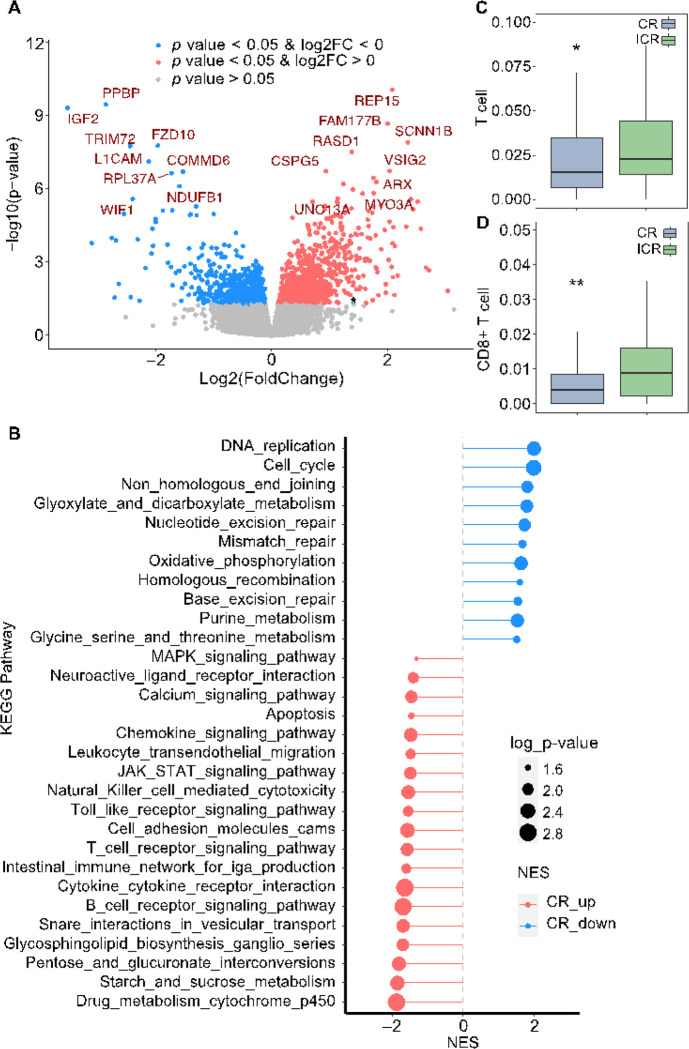
Differences in transcriptomic profile and immune cell infiltration between CR and ICR to nCRT. **A:** Volcano plot displaying differentially expressed genes (DEGs) between complete responders (CR) and incomplete responders (ICR). Genes with p < 0.05 and increased expression are shown in red, decreased expression in blue, and non-significant changes in gray. **B**: Pathway Enrichment Analysis. The chart displays significantly enriched KEGG pathways based on differentially expressed genes (DEGs) in complete responders (CR). Pathways more active in CR are indicated in blue (CR down) and those less active in red (CR up). The size of the dot correlates with the −log10 p-value, indicating the statistical significance of the enrichment. **C**, **D**: Box plots comparing the infiltration levels of T cells (**C**) and CD8+ T cells (**D**) within tumor microenvironments between CR (n=36) and ICR (n=86) groups. Statistical significance was determined using the Mann-Whitney U test, with * indicating p < 0.05 and ** indicating p < 0.01.

To compare the transcriptomic differences between CR and ICR tumors, we merged the RNA sequencing data from 114 patients from the MSK cohort and 7 patients from the UM cohort. We identified 678 downregulated and 1018 upregulated genes in CR tumors (*p*-value < 0.05 and log2FC > 0) (Fig. 4A). Consistent with the study from Chatila *et.al* [16], *IGF2* and *L1CAM* were distinctly overexpressed in ICR tumors. Gene Set Enrichment Analysis (GSEA) revealed that genes with relatively low expression in CR tumors were significantly enriched in several DNA repair-associated pathways, including non-homologous end joining (NHEJ), NER, MMR, homologous recombination (HR), and BER pathways (Fig. 4B). This is consistent with our mutation data analysis where DNA repair genes were predominantly mutated, leading to DNA repair deficiency. CR tumors also demonstrated overexpression of numerous genes that were significantly enriched in immune-related pathways (Fig. 4B, **Supplementary Fig. 3**). In addition to the association of tumorigenic pathways with mutation data, gene expression analysis suggests that increased immune function in CR tumors may be critical to nCRT as well.

Emerging evidence shows that tumor-infiltrating immune cells (TICs) are associated with sensitivity to nCRT [48, 49]. To investigate the differences in TICs between CR and ICR tumors, we employed TIMER [32] and subsequently assessed the differential fractions/enrichment scores. We observed that CR tumors manifested a significantly reduced infiltration of T cells, particularly CD8 + T cells (Mann Whitney U test, *p*-value < 0.05) (Fig. 4C–D). In a contrasting observation, although not reaching statistical significance (Mann Whitney U test, *p*-value > 0.05), ICR tumors exhibited lower fractions/enrichment scores across a spectrum of immune cells, including B cells, Neutrophil, M2 macrophages, dendritic cells, neutral killer (NK) cells, Neutrophils, and monocytes (**Supplementary Data 2**).

### Predictors of complete response to neoadjuvant therapy in rectal cancer

To elucidate both clinical and genomic predictors of complete response (CR), we selected patients with clinical stage II and III who received neoadjuvant therapy and for whom targeted or whole exome DNA sequencing data was available. This resulted in 233 ICR and 101 CR patients for analysis. The bivariate analysis revealed that the only clinical variable that differed between CR and ICR patients was median tumor size (CR 4.2 cm (Q1-Q3) 3.2–5.2) vs ICR (4.7 cm (3.8–6.0)) (**Supplementary Table 1**). Treatment group did not predict whether a patient went on to be a CR or ICR (**Supplementary Table 1**). We next assessed whether presence of particular non-synonymous deleterious mutations differed between CR and ICR and identified 10 alterations that were more common in CR patients with p-values of 0.1 or less (**Supplementary Table 3**). Network analysis indicated interactions between all 10 genes (**Supplementary Table 4**). These interactions, such as co-expression, were identified through curated databases (**Supplementary Fig. 4**). Because CR patients had more co-occurring mutations when the genomic analysis was performed (Fig. 3D–E), we did bivariate comparisons between all identified co-occurring mutations in CR and ICR patients and identified a subset of 95 that were more common in CR patients with p-values < 0.1 (**Supplementary Data 3**). Next, we fit a logistic regression model for CR using tumor size (cm), clinical stage, a mutation in any of the 10 network genes, and number of co-occurring mutations grouped as 0, 1–2, or 3 or more co-occurring mutations (**Supplementary Data 4**). After selecting the model with the highest R^2^ Tjur and lowest AIC, we observed that predictors of CR included smaller tumor size (OR: 0.79, 95%CI: 0.66–0.94), mutation of any of the network genes (OR: 2.57, 95%CI: 1.37–4.77), and presence of more co-occurring mutations (likelihood ratio test p-value < 0.001). Compared to patients with zero co-occurring mutations, those with 1 to 2 mutations exhibited a 1.57 (95%CI: 0.67–3.51) higher odds of CR and those with 3 or more co-occurring mutations showed an even greater 8.47 (95%CI: 2.74–32.26) times higher odds of CR (Table 1).

### Genomic and molecular determinants of incomplete response to nCRT with subsequent recurrence

**Figure 5:**
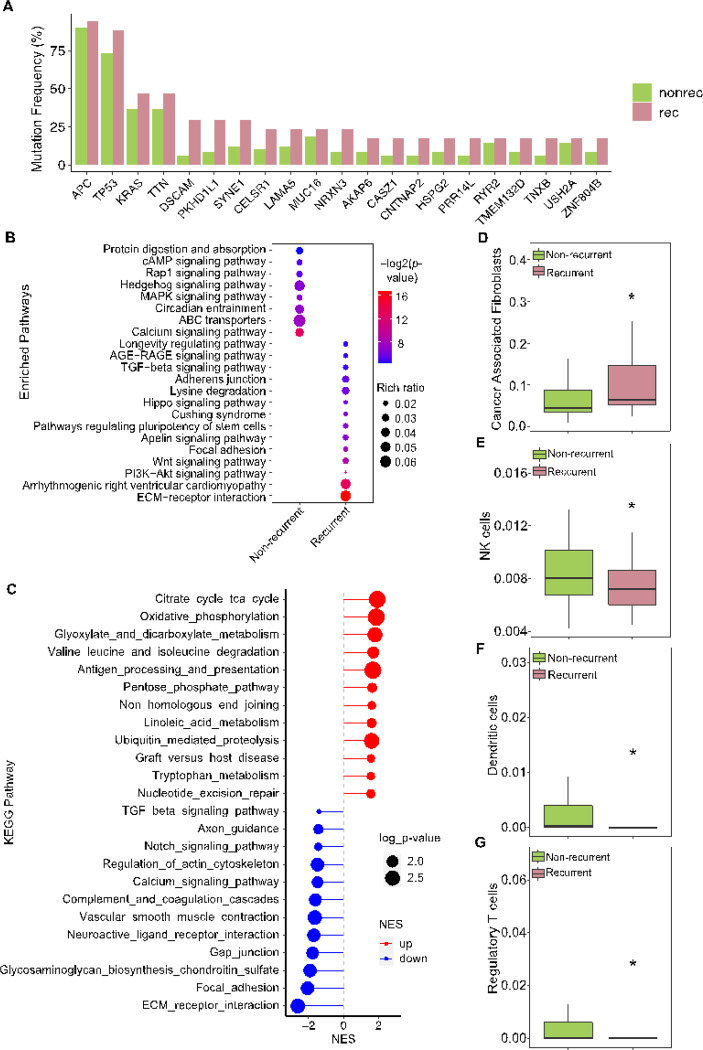
Genomic and immune landscape differences associated with recurrence in incomplete responders to nCRT. **A:** Bar plot displaying the frequency of mutations in genes for ICR tumors, differentiating between recurrent (rec, n=17) and non-recurrent (nonrec, n=49) cases. **B**: Enrichment analysis of KEGG pathways comparing frequently mutated genes in recurrent versus non-recurrent ICR tumors, with pathway significance denoted by color intensity and dot size representing the rich ratio. **C**: Enrichment plot illustrating the significantly enriched pathways for differentially expressed genes (DEGs) between recurrent and non-recurrent ICR tumors, with upregulated pathways in red and downregulated in blue. **D**-**G**: Box plots quantifying the relative abundance of cancer-associated fibroblasts (**D**), NK cells (**E**), dendritic cells (**F**), and regulatory T cells (**G**) in recurrent (n=23) compared to non-recurrent ICR tumors (n=53). Statistical significance determined by Mann-Whitney U test, with * indicating p < 0.05.

Given that approximately one-third of ICR tumors subsequently recurred, we explored the molecular features associated with recurrence in these pre-treatment biopsy samples, aiming to enhance our understanding and explore potential strategies specifically to target these recurrent ICR tumors. Although recurrent ICR patients had significantly (the log rank test, *p*-value < 0.0001) poorer survival (**Supplementary Fig. 5A**), there was no significant difference in their tumor mutation burden, altered genome fractions, or MATH scores [50] (**Supplementary Fig. 5B-D**). However, these tumors that later reoccurred exhibited a higher frequency of mutations in cancer driver genes, including *APC, TP53, KRAS*, and *TTN* (Fig. 5A). Enrichment analysis revealed these tumors had more mutations in pathways related to TGF-ß signaling, ECM-receptor interaction, and cell-cell interaction (Fig. 5B). In addition, gene expression profiles indicated a downregulation of cell-cell interaction genes in recurrent ICR tumors (Fig. 5C). Moreover, more Cancer Associated Fibroblasts (CAFs), known to influence colorectal cancer (CRC) metastasis [51], were detected in the recurrent ICR tumors (Fig. 5D). Consistent with enriched mutations and decreased expression of cell-cell interaction genes, targeting CAFs may be a potential therapeutic strategy to prevent metastasis in CRC [52, 53]. Additionally, recurrent tumors demonstrated reduced infiltration of NK cells, dendritic cells, and Regulatory T Cells (Tregs) (Fig. 5E–G).

### Characteristics of subgroups of the incomplete responders

**Figure 6:**
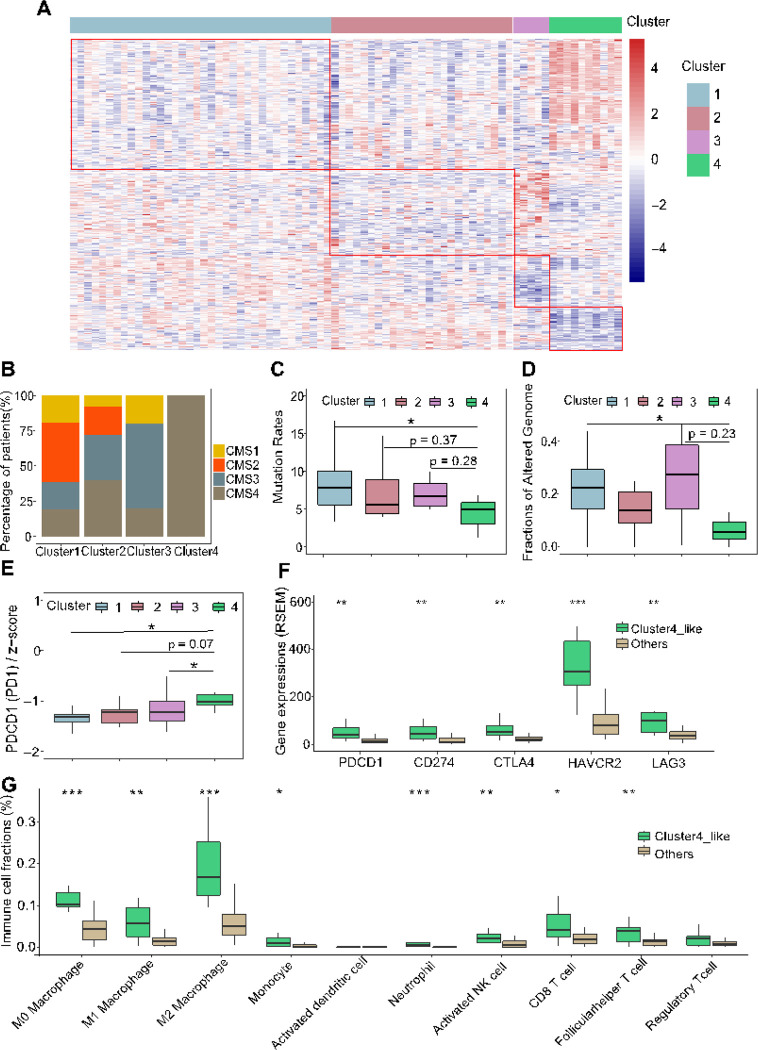
Gene expression profiling reveals an immune-active tumor subtype in incomplete responders associated with increased resistance and pooper prognosis post-neoadjuvant therapy. **A:** Heatmap showing distinct gene expression profiles across four identified subgroups of ICR tumors (n=76; Cluster1: n=36, Cluster2: n=25, Cluster3: n=5, Cluster4: n=10). **B**: Distribution of Consensus Molecular Subtype (CMS) classifications within each ICR tumor subgroup. **C-D**: Boxplots detailing comparison of mutation rate (**C**) and fraction of altered genome (**D**) among the four ICR subgroups (Mann Whitney U test, p < 0.05, total n=39, Cluster 1: n=27, Cluster 2: n=15, Cluster 3: n=4, Cluster 4: n=3). **E**: Boxplot comparing PD1 gene expression across the four ICR subgroups (Mann Whitney U test, p < 0.05, n=76, Cluster 1: n=36, Cluster 2: n=25, Cluster 3: n=5, Cluster 4: n=10). **F**: Comparisons of immune checkpoint genes (PD1, PD-L1, CTLA4, HAVCR2, LAG3) expression in the TCGA READ dataset, with a focus on contrasting Cluster 4-like with other clusters (Mann Whitney U test, *p < 0.05, **p<0.01, ***p<0.001, n=41, Cluster 4-like: n=10, Others: n=31). **G**: Assessment of Immune cell fractions within the TCGA READ dataset, showcasing differences between Cluster 4-like and other clusters (Mann Whitney U test, *p < 0.05, **p<0.01, ***p<0.001, n=41, Cluster 4-like: n=10, Others: n=31).

Given the evidence [54] that gene expression has a higher predictive capacity for drug response than other factors such as mutation signature, copy number variation, and methylation, we aimed to identify ICR patient subgroups based on gene expression patterns. Our objective was to understand the molecular characteristics of these subgroups, potentially guiding alternative therapeutic approaches. Through unsupervised hierarchical clustering of 76 ICR patients with whole exome and transcriptome sequencing and outcome data (**Supplementary Data 5**), we identified four groups with distinct gene expression profiles (Fig. 6A, **Supplementary Fig. 6A**).

Cluster 1 tumors (n = 36) exhibited characteristics similar to CR tumors, marked by elevated mutation rates, enhanced cell-cell interaction, and diminished expression of DNA repair genes (**Supplementary Fig. 6B**). Cluster 2 tumors (n = 25) showed efficient DNA repair capabilities, albeit with reduced expression of pathways associated with drug metabolism (**Supplementary Fig. 6C**). Cluster 3 (n = 5) exhibited a significant upregulation of cytokine and chemokine signaling pathways (**Supplementary Fig. 6D**), suggesting potential opportunities for cytokine-based therapeutic interventions [55]. Interestingly, all tumors in Cluster 4 (n = 10) were CMS4 subtype (Fig. 6B), which is associated with poorer survival [56, 57]. This tumor subset exhibited a higher proportion of metastatic patients (25% in Cluster 1 compared to 40% in Cluster 4, **Supplementary Fig. 6E**). Additionally, these tumors displayed distinct genomic alternations, including decreased mutation rates and lower fraction of altered genomes (Fig. 6C–D), along with lower expression of several cell-cell interaction pathways (**Supplementary Fig. 6F**). Among the top 50 DEGs between tumors in Cluster 4 and other clusters, we identified 4 genes (*DUOXA2, DUOX2, SSH1, FRMD6*) that were significantly associated with worse survival (**Supplementary Fig. 6G-J**). Previous studies have reported that *DUOX2* and *SSH1* exhibited significantly higher expression and promoted the progression and metastasis of CRC [58, 59]. Additionally, *FRMD6*, which is an upstream regulator of Hippo signaling, serves as a marker of the CMS4 subgroup in CRC [60, 61].

Furthermore, the immune microenvironment of Cluster 4 tumors was characterized by increased infiltration of various immune cells, including neutrophils, monocytes, macrophages, activated myeloid dendritic cells, naive CD4 + T cell, as well as Tregs cells (**Supplementary Fig. 6K-P**). The immune-rich microenvironment of Cluster 4 may indicate that there is an opportunity for immunotherapy for this specific subset of tumors. Of note, we observed significant overexpression of *PDCD1* (PD-1) in Cluster 4 (Fig. 6E), highlighting its potential as a therapeutic target [62, 63].

To validate these findings, we applied the differential expression signature between Cluster 4 and other clusters to 42 rectum adenocarcinoma (READ) samples in TCGA. We identified that a subgroup, termed Cluster4-like, demonstrated a similar expression profile to Cluster 4 (**Supplementary Fig. 6Q-R**). Interestingly, the tumors in the Cluster4-like group (n = 10) also had poor outcomes, with 80% being CMS4 subtype (**Supplementary Fig. 6S**). Immune checkpoint genes, including PD-1, *CD274* (PD-L1), *CTLA4, HAVCR2* (TIM3), and *LAG3*, were overexpressed in Cluster4-like tumors (Fig. 6F). These tumors also displayed increased immune cell infiltration (Fig. 6G). These differences mirrored Cluster 4 characteristics. Collectively, our findings suggest that greater attention should be given to the subsets of patients with high immune cell infiltration and overexpression of immune checkpoint genes, as these patients might benefit from immune checkpoint inhibitors (ICIs).

## Discussion

Rectal cancer treatment is evolving. Recent clinical studies have focused on optimizing treatment by altering the order of chemotherapy and chemoradiotherapy relative to surgery and have identified patients where organ preservation appears feasible which will lead to decreased surgery. Yet, molecular predictors of treatment response remain unidentified [4]. In this current study, we have gathered public and institutional genomic data from pre-treatment biopsies of patients undergoing chemoradiotherapy, followed by clinical response assessment or surgery. Our in-depth comparative analysis sought to answer the 2 most fundamental clinical questions in the care of rectal cancer patients: how CR and ICR patient tumors differ prior to treatment and how tumors from patients who later will recur differ from those who don’t. We identified multiple important differences, some of which are potentially targetable. We utilized advanced bioinformatics approaches for these comparisons, including the use of the propensity score weighting to balance confounding factors that may have been associated with genetic changes, such as BMI, age, gender, tumor size, and N and T stages.

Our comprehensive analysis has identified important molecular and clinical factors influencing the response to neoadjuvant chemoradiotherapy (nCRT). We identified that ICR patients presented with larger tumor sizes and go on to have higher recurrence rates compared to CR patients. CR tumors demonstrated a higher mutation burden (TMB) and frequent mutations in genes linked to DNA repair pathways, while ICR tumors had higher mutation frequencies in oncogenic signaling pathways. Furthermore, CR tumors showed a higher rate of co-occurring mutations, enrichment in DNA repair alterations and DNA damage response networks. Transcriptomic analysis revealed that ICR tumors overexpressed DNA repair genes, while CR tumors have increases in genes in immune pathways. The immune microenvironment of CR tumors had significantly fewer infiltrating T cells, particularly CD8 + T cells. Using logistic regression analysis, we identified potential CR predictors for future studies, including alterations in specific gene networks and multiple co-occurring mutations. Our study also revealed unique characteristics for ICR tumors that later recurred, including increased TGF-ß signaling and impaired cell-cell interaction. Lastly, we identified four distinct ICR subgroups based on gene expression, each suggesting different therapeutic strategies. Specifically, the Cluster-4 group, with a CMS-like tumor expression profile, demonstrated the greatest resistance to nCRT and most immune infiltration, suggesting the potential for targeted checkpoint inhibitor therapy to improve the outcome in this challenging patient subgroup.

Previous retrospective studies have reported that deficiency in mismatch repair (MSI-H status) have similar complete response rates to nCRT as mismatch proficient tumors but may have improved downstaging with treatment [64, 65]. In our study, we observed that mutations in multiple DNA repair pathways were enriched in CR patients. This was not due to differences in MSI-H tumors which were only about 2% of tumors in both CR and ICR groups. The mutations we identified in DNA repair align with decreased expression of DNA repair mRNAs. In contrast, ICR tumors exhibit a increases in DNA damage repair gene expression. Our findings underscore that defective DNA repair mechanisms, including dMMR, dNER, dBER, and dHRR, relate to a complete response to nCRT. Prior studies have not assessed the spectrum of DNA repair pathways as we have done here. The activation of DNA repair in tumor cells likely is in response to DNA damage induced by chemoradiotherapy and when the repair is effective, this will result in ICR. Moreover, a higher frequency of co-occurring mutations in CR tumors suggests the potential of synthetic lethality, a condition where concurrent genetic events lead to cell death [66]. Our data indicate that this phenomenon is strongly correlated with the response to nCRT, again indicating that improved response to treatment aligns with an inability to overcome tumor damage from treatment. Our study suggests that evaluating mutation status in predictor genes and specific co-occurring mutations may predict CR status, potentially guiding surgical decision making.

Rectal cancer is a complex and molecularly heterogeneous disease [67]. For ICR tumors, our objective was to identify subsets that may be responsive to alternative treatments. We first investigated the molecular features of recurrent ICR tumors. Compared to nonrecurrent ICR tumors, recurrent counterparts had no significant differences in TMB or genome alterations (**Supplementary Fig. 5B-C**). However, ICR tumors which later recurred, displayed a higher frequency of mutations in cell-cell interaction pathways, including focal adhesion, adherens junction, TGF-ß signaling, and ECM-receptor interaction. Moreover, the expression data found diminished activity in these pathways, suggesting impaired cell-cell interaction is characteristic of these tumors. Therefore, strategies targeting this dysregulation may benefit patients with these alterations which we identified in ICR tumors that went on to recur. Interestingly, these differences are already identifiable in pre-treatment biopsies indicating that tumors harbor alterations which cause subsequent metastasis even at diagnosis.

Subsequently, we identified a subset of ICR tumors using gene expression profiles and validated these using TCGA data. This patient subset, characterized by poor outcomes, exhibited a reduced mutation burden and genomic alteration but displayed significantly increased immune cell infiltration. Moreover, the over-expression of immune checkpoint mRNAs suggests a potential responsiveness to immune checkpoint inhibitors.

## Figures and Tables

**Table 1:** Logistic regression model coefficients for prediction of complete response (CR) to treatment

*Predictors*	*Odds Ratios*	*95% CI*	*p* value
Tumor size (cm)	0.79	0.66–0.94	**<0.01**
AJCC classification			
2	Ref.		
3	0.71	0.38–1.35	0.28
Number of selected co-occurring mutations			
0	Ref.		
1–2	1.57	0.67–3.51	0.29
3 or more	8.47	2.74–32.26	**<0.001**
Mutations in selected genes			
No	Ref.		
Yes	2.57	1.37–4.77	**<0.01**
*Observations*	*314*		
*R*^*2*^ *Tjur*	*0.14*		
*AIC*	*349.72*		

CI: Confidence interval; AIC: Akaike Information Criterion

## Data Availability

All data are available from the corresponding authors upon reasonable request. For RNA-seq data has been deposited in the Gene Expression Omnibus database under accession GSE242786, and the public dataset under accession GSE209746.

## References

[1] Siegel RL, Miller KD, Fuchs HE, Jemal A: Cancer statistics, 2022. CA Cancer J Clin 2022, 72(1):7–33. 35020204 10.3322/caac.21708

[2] Benson AB, Venook AP, Al-Hawary MM, Azad N, Chen Y-J, Ciombor KK, Cohen S, Cooper HS, Deming D, Garrido-Laguna I : Rectal Cancer, Version 2.2022, NCCN Clinical Practice Guidelines in Oncology. J Natl Compr Canc Netw 2022, 20(10):1139–1167. 36240850 10.6004/jnccn.2022.0051

[3] Kamran SC, Lennerz JK, Margolis CA, Liu D, Reardon B, Wankowicz SA, Van Seventer EE, Tracy A, Wo JY, Carter SL : Integrative Molecular Characterization of Resistance to Neoadjuvant Chemoradiation in Rectal Cancer. Clin Cancer Res 2019, 25(18):5561–5571. 31253631 10.1158/1078-0432.CCR-19-0908 PMC6744983

[4] Garcia-Aguilar J, Patil S, Gollub MJ, Kim JK, Yuval JB, Thompson HM, Verheij FS, Omer DM, Lee M, Dunne RF : Organ Preservation in Patients With Rectal Adenocarcinoma Treated With Total Neoadjuvant Therapy. J Clin Oncol 2022, 40(23):2546–2556. 35483010 10.1200/JCO.22.00032 PMC9362876

[5] Maas M, Nelemans PJ, Valentini V, Das P, Rödel C, Kuo L-J, Calvo FA, García-Aguilar J, Glynne-Jones R, Haustermans K : Long-term outcome in patients with a pathological complete response after chemoradiation for rectal cancer: a pooled analysis of individual patient data. Lancet Oncol 2010, 11(9):835–844. 20692872 10.1016/S1470-2045(10)70172-8

[6] García-Aguilar J, Hernandez de Anda E, Sirivongs P, Lee S-H, Madoff RD, Rothenberger DA: A pathologic complete response to preoperative chemoradiation is associated with lower local recurrence and improved survival in rectal cancer patients treated by mesorectal excision. Dis Colon Rectum 2003, 46(3):298–304. 12626903 10.1007/s10350-004-6545-x

[7] Zhou C, Wang K, Zhang X, Xiao Y, Yang C, Wang J, Qu F, Wang X, Liu M, Gao C : Assessing the predictive value of clinical factors to pathological complete response for locally advanced rectal cancer: An analysis of 124 patients. Front Oncol 2023, 13:1125470. 37064150 10.3389/fonc.2023.1125470 PMC10102576

[8] Qin C-J, Song X-M, Chen Z-H, Ren X-Q, Xu K-W, Jing H, He Y-L: XRCC2 as a predictive biomarker for radioresistance in locally advanced rectal cancer patients undergoing preoperative radiotherapy. Oncotarget 2015, 6(31):32193–32204. 26320178 10.18632/oncotarget.4975 PMC4741669

[9] Timudom K, Akaraviputh T, Chinswangwatanakul V, Pongpaibul A, Korpraphong P, Petsuksiri J, Ithimakin S, Trakarnsanga A: Predictive significance of cancer related-inflammatory markers in locally advanced rectal cancer. World J Gastrointest Surg 2020, 12(9):390–396. 33024513 10.4240/wjgs.v12.i9.390 PMC7520570

[10] Kim S, Yeo M-K, Kim J-S, Kim J-Y, Kim K-H: Elevated CXCL12 in the plasma membrane of locally advanced rectal cancer after neoadjuvant chemoradiotherapy: a potential prognostic marker. J Cancer 2022, 13(1):162–173. 34976180 10.7150/jca.64082 PMC8692683

[11] Momma T, Okayama H, Kanke Y, Fukai S, Onozawa H, Fujita S, Sakamoto W, Saito M, Ohki S, Kono K: Validation of Gene Expression-Based Predictive Biomarkers for Response to Neoadjuvant Chemoradiotherapy in Locally Advanced Rectal Cancer. Cancers 2021, 13(18). 10.3390/cancers13184642 PMC8467397 34572869

[12] Garcia-Aguilar J, Chen Z, Smith DD, Li W, Madoff RD, Cataldo P, Marcet J, Pastor C: Identification of a biomarker profile associated with resistance to neoadjuvant chemoradiation therapy in rectal cancer. Ann Surg 2011, 254(3):486–492; discussion 492 – 483. 21865946 10.1097/SLA.0b013e31822b8cfa PMC3202983

[13] De Mattia E, Polesel J, Mezzalira S, Palazzari E, Pollesel S, Toffoli G, Cecchin E: Predictive and prognostic value of oncogene mutations and microsatellite instability in locally-advanced rectal cancer treated with neoadjuvant radiation-based therapy: A systematic review and meta-analysis. Cancers 2023, 15(5):1469. 36900260 10.3390/cancers15051469 PMC10001009

[14] Yang J, Lin Y, Huang Y, Jin J, Zou S, Zhang X, Li H, Feng T, Chen J, Zuo Z : Genome landscapes of rectal cancer before and after preoperative chemoradiotherapy. Theranostics 2019, 9(23):6856–6866. 31660073 10.7150/thno.37794 PMC6815957

[15] Cancer Genome Atlas N: Comprehensive molecular characterization of human colon and rectal cancer. Nature 2012, 487(7407):330–337. 22810696 10.1038/nature11252 PMC3401966

[16] Chatila WK, Kim JK, Walch H, Marco MR, Chen C-T, Wu F, Omer DM, Khalil DN, Ganesh K, Qu X : Genomic and transcriptomic determinants of response to neoadjuvant therapy in rectal cancer. Nat Med 2022, 28(8):1646–1655. 35970919 10.1038/s41591-022-01930-z PMC9801308

[17] Van der Auwera GA, Carneiro MO, Hartl C, Poplin R, Del Angel G, Levy-Moonshine A, Jordan T, Shakir K, Roazen D, Thibault J : From FastQ data to high confidence variant calls: the Genome Analysis Toolkit best practices pipeline. Curr Protoc Bioinformatics 2013, 43(1110):11.10.11–11.10.33. 10.1002/0471250953.bi1110s43 PMC4243306 25431634

[18] Kim S, Scheffler K, Halpern AL, Bekritsky MA, Noh E, Källberg M, Chen X, Kim Y, Beyter D, Krusche P : Strelka2: fast and accurate calling of germline and somatic variants. Nat Methods 2018, 15(8):591–594. 30013048 10.1038/s41592-018-0051-x

[19] McLaren W, Gil L, Hunt SE, Riat HS, Ritchie GRS, Thormann A, Flicek P, Cunningham F: The Ensembl Variant Effect Predictor. Genome Biol 2016, 17(1):122. 27268795 10.1186/s13059-016-0974-4 PMC4893825

[20] Kandoth C: mskcc/vcf2maf: vcf2maf v1.6.19 doi:10.5281/zenodo.593251. In.; 2020.

[21] Li F, Morgan KL, Zaslavsky AM: Balancing covariates via propensity score weighting. J Am Stat Assoc 2018, 113(521):390–400.

[22] Li L, Greene T: A weighting analogue to pair matching in propensity score analysis. Int J Biostat 2013, 9(2):215–234. 23902694 10.1515/ijb-2012-0030

[23] Lawrence MS, Stojanov P, Mermel CH, Robinson JT, Garraway LA, Golub TR, Meyerson M, Gabriel SB, Lander ES, Getz G: Discovery and saturation analysis of cancer genes across 21 tumour types. Nature 2014, 505(7484):495–501. 24390350 10.1038/nature12912 PMC4048962

[24] Kuleshov MV, Jones MR, Rouillard AD, Fernandez NF, Duan Q, Wang Z, Koplev S, Jenkins SL, Jagodnik KM, Lachmann A : Enrichr: a comprehensive gene set enrichment analysis web server 2016 update. Nucleic Acids Res 2016, 44(W1):W90–97. 27141961 10.1093/nar/gkw377 PMC4987924

[25] Mayakonda A, Lin D-C, Assenov Y, Plass C, Koeffler HP: Maftools: efficient and comprehensive analysis of somatic variants in cancer. Genome Res 2018, 28(11):1747–1756. 30341162 10.1101/gr.239244.118 PMC6211645

[26] Szklarczyk D, Gable AL, Lyon D, Junge A, Wyder S, Huerta-Cepas J, Simonovic M, Doncheva NT, Morris JH, Bork P : STRING v11: protein-protein association networks with increased coverage, supporting functional discovery in genome-wide experimental datasets. Nucleic Acids Res 2019, 47(D1):D607–D613. 30476243 10.1093/nar/gky1131 PMC6323986

[27] Shannon P, Markiel A, Ozier O, Baliga NS, Wang JT, Ramage D, Amin N, Schwikowski B, Ideker T: Cytoscape: a software environment for integrated models of biomolecular interaction networks. Genome Res 2003, 13(11):2498–2504. 14597658 10.1101/gr.1239303 PMC403769

[28] Dobin A, Davis CA, Schlesinger F, Drenkow J, Zaleski C, Jha S, Batut P, Chaisson M, Gingeras TR: STAR: ultrafast universal RNA-seq aligner. Bioinformatics 2013, 29(1):15–21. 23104886 10.1093/bioinformatics/bts635 PMC3530905

[29] Anders S, Pyl PT, Huber W: HTSeq—a Python framework to work with high-throughput sequencing data. Bioinformatics 2014, 31(2):166–169. 25260700 10.1093/bioinformatics/btu638 PMC4287950

[30] Love MI, Huber W, Anders S: Moderated estimation of fold change and dispersion for RNA-seq data with DESeq2. Genome Biol 2014, 15(12):550. 25516281 10.1186/s13059-014-0550-8 PMC4302049

[31] Subramanian A, Tamayo P, Mootha VK, Mukherjee S, Ebert BL, Gillette MA, Paulovich A, Pomeroy SL, Golub TR, Lander ES : Gene set enrichment analysis: a knowledge-based approach for interpreting genome-wide expression profiles. Proc Natl Acad Sci U S A 2005, 102(43):15545–15550. 16199517 10.1073/pnas.0506580102 PMC1239896

[32] Li T, Fan J, Wang B, Traugh N, Chen Q, Liu JS, Li B, Liu XS: TIMER: A Web Server for Comprehensive Analysis of Tumor-Infiltrating Immune Cells. Cancer Res 2017, 77(21):e108–e110. 29092952 10.1158/0008-5472.CAN-17-0307 PMC6042652

[33] Newman AM, Liu CL, Green MR, Gentles AJ, Feng W, Xu Y, Hoang CD, Diehn M, Alizadeh AA: Robust enumeration of cell subsets from tissue expression profiles. Nat Methods 2015, 12(5):453–457. 25822800 10.1038/nmeth.3337 PMC4739640

[34] Finotello F, Mayer C, Plattner C, Laschober G, Rieder D, Hackl H, Krogsdam A, Loncova Z, Posch W, Wilflingseder D : Molecular and pharmacological modulators of the tumor immune contexture revealed by deconvolution of RNA-seq data. Genome Med 2019, 11(1):34. 31126321 10.1186/s13073-019-0638-6 PMC6534875

[35] Aran D, Hu Z, Butte AJ: xCell: digitally portraying the tissue cellular heterogeneity landscape. Genome Biol 2017, 18(1):220. 29141660 10.1186/s13059-017-1349-1 PMC5688663

[36] Becht E, Giraldo NA, Lacroix L, Buttard B, Elarouci N, Petitprez F, Selves J, Laurent-Puig P, Sautès-Fridman C, Fridman WH : Estimating the population abundance of tissue-infiltrating immune and stromal cell populations using gene expression. Genome Biol 2016, 17(1):218. 27765066 10.1186/s13059-016-1070-5 PMC5073889

[37] Racle J, de Jonge K, Baumgaertner P, Speiser DE, Gfeller D: Simultaneous enumeration of cancer and immune cell types from bulk tumor gene expression data. Elife 2017, 6. 10.7554/eLife.26476 PMC5718706 29130882

[38] Menard S: Applied Logistic Regression Analysis, 2 edn. Christchurch, New Zealand: Sage Publications; 2018.

[39] Robin X, Turck N, Hainard A, Tiberti N, Lisacek F, Sanchez J-C, Müller M: pROC: an open-source package for R and S + to analyze and compare ROC curves. BMC Bioinformatics 2011, 12(1):77. 21414208 10.1186/1471-2105-12-77 PMC3068975

[40] Smith JJ, Strombom P, Chow OS, Roxburgh CS, Lynn P, Eaton A, Widmar M, Ganesh K, Yaeger R, Cercek A : Assessment of a Watch-and-Wait Strategy for Rectal Cancer in Patients With a Complete Response After Neoadjuvant Therapy. JAMA Oncol 2019, 5(4):e185896. 30629084 10.1001/jamaoncol.2018.5896 PMC6459120

[41] Stockton JD, Tee L, Whalley C, James J, Dilworth M, Wheat R, Nieto T, Consortium SC, Geh I, Barros-Silva JD : Complete response to neoadjuvant chemoradiotherapy in rectal cancer is associated with RAS/AKT mutations and high tumour mutational burden. Radiat Oncol 2021, 16(1):129. 34256782 10.1186/s13014-021-01853-y PMC8278688

[42] Mina M, Raynaud F, Tavernari D, Battistello E, Sungalee S, Saghafinia S, Laessle T, Sanchez-Vega F, Schultz N, Oricchio E : Conditional Selection of Genomic Alterations Dictates Cancer Evolution and Oncogenic Dependencies. Cancer Cell 2017, 32(2):155–168.e156. 28756993 10.1016/j.ccell.2017.06.010

[43] Kuzmin E, VanderSluis B, Wang W, Tan G, Deshpande R, Chen Y, Usaj M, Balint A, Mattiazzi Usaj M, van Leeuwen J : Systematic analysis of complex genetic interactions. Science 2018, 360(6386). 10.1126/science.aao1729 PMC6215713 29674565

[44] Giannakis M, Hodis E, Jasmine Mu X, Yamauchi M, Rosenbluh J, Cibulskis K, Saksena G, Lawrence MS, Qian ZR, Nishihara R : RNF43 is frequently mutated in colorectal and endometrial cancers. Nat Genet 2014, 46(12):1264–1266. 25344691 10.1038/ng.3127 PMC4283570

[45] Danielsen SA, Lind GE, Bjørnslett M, Meling GI, Rognum TO, Heim S, Lothe RA: Novel mutations of the suppressor gene PTEN in colorectal carcinomas stratified by microsatellite instability- and TP53 mutation-status. Hum Mutat 2008, 29(11):E252–262. 18781614 10.1002/humu.20860

[46] Kurose K, Gilley K, Matsumoto S, Watson PH, Zhou X-P, Eng C: Frequent somatic mutations in PTEN and TP53 are mutually exclusive in the stroma of breast carcinomas. Nat Genet 2002, 32(3):355–357. 12379854 10.1038/ng1013

[47] Donehower LA, Soussi T, Korkut A, Liu Y, Schultz A, Cardenas M, Li X, Babur O, Hsu T-K, Lichtarge O : Integrated Analysis of TP53 Gene and Pathway Alterations in The Cancer Genome Atlas. Cell Rep 2019, 28(5):1370–1384.e1375. 31365877 10.1016/j.celrep.2019.07.001 PMC7546539

[48] Orhan A, Khesrawi F, Tvilling Madsen M, Peuliche Vogelsang R, Dohrn N, Kanstrup Fiehn A-M, Gögenur I: Tumor-Infiltrating Lymphocytes as Biomarkers of Treatment Response and Long-Term Survival in Patients with Rectal Cancer: A Systematic Review and Meta-Analysis. Cancers 2022, 14(3). 10.3390/cancers14030636 PMC8833320 35158905

[49] Yasuda K, Nirei T, Sunami E, Nagawa H, Kitayama J: Density of CD4(+) and CD8(+) T lymphocytes in biopsy samples can be a predictor of pathological response to chemoradiotherapy (CRT) for rectal cancer. Radiat Oncol 2011, 6:49. 21575175 10.1186/1748-717X-6-49 PMC3120676

[50] Rajput A, Bocklage T, Greenbaum A, Lee J-H, Ness SA: Mutant-Allele Tumor Heterogeneity scores correlate with risk of metastases in colon cancer. Clin Colorectal Cancer 2017, 16(3):e165–e170. 28073683 10.1016/j.clcc.2016.11.004 PMC5441963

[51] Zhong B, Cheng B, Huang X, Xiao Q, Niu Z, Chen Y-F, Yu Q, Wang W, Wu X-J: Colorectal cancer-associated fibroblasts promote metastasis by up-regulating LRG1 through stromal IL-6/STAT3 signaling. Cell Death Dis 2021, 13(1):16. 34930899 10.1038/s41419-021-04461-6 PMC8688517

[52] Kang S, Tanaka T, Narazaki M, Kishimoto T: Targeting Interleukin-6 Signaling in Clinic. Immunity 2019, 50(4):1007–1023. 30995492 10.1016/j.immuni.2019.03.026

[53] Jeong K-Y: Inhibiting focal adhesion kinase: A potential target for enhancing therapeutic efficacy in colorectal cancer therapy. World J Gastrointest Oncol 2018, 10(10):290–292. 30364839 10.4251/wjgo.v10.i10.290 PMC6198301

[54] Levatić J, Salvadores M, Fuster-Tormo F, Supek F: Mutational signatures are markers of drug sensitivity of cancer cells. Nat Commun 2022, 13(1):2926. 35614096 10.1038/s41467-022-30582-3 PMC9132939

[55] Propper DJ, Balkwill FR: Harnessing cytokines and chemokines for cancer therapy. Nat Rev Clin Oncol 2022, 19(4):237–253. 34997230 10.1038/s41571-021-00588-9

[56] Thanki K, Nicholls ME, Gajjar A, Senagore AJ, Qiu S, Szabo C, Hellmich MR, Chao C: Consensus Molecular Subtypes of Colorectal Cancer and their Clinical Implications. Int Biol Biomed J 2017, 3(3):105–111. 28825047 PMC5557054

[57] Guinney J, Dienstmann R, Wang X, de Reyniès A, Schlicker A, Soneson C, Marisa L, Roepman P, Nyamundanda G, Angelino P : The consensus molecular subtypes of colorectal cancer. Nat Med 2015, 21(11):1350–1356. 26457759 10.1038/nm.3967 PMC4636487

[58] Song X, Xie D, Xia X, Tan F, Pei Q, Li Y, Zhou Z, Zhou Y, Li C, Wang K : Role of SSH1 in colorectal cancer prognosis and tumor progression. J Gastroenterol Hepatol 2020, 35(7):1180–1188. 32020663 10.1111/jgh.15001

[59] Zhang X, Han J, Feng L, Zhi L, Jiang D, Yu B, Zhang Z, Gao B, Zhang C, Li M : DUOX2 promotes the progression of colorectal cancer cells by regulating the AKT pathway and interacting with RPL3. Carcinogenesis 2021, 42(1):105–117. 32531052 10.1093/carcin/bgaa056 PMC7877561

[60] De Sousa E Melo F, Wang X, Jansen M, Fessler E, Trinh A, de Rooij LPMH, de Jong JH, de Boer OJ, van Leersum R, Bijlsma MF : Poor-prognosis colon cancer is defined by a molecularly distinct subtype and develops from serrated precursor lesions. Nat Med 2013, 19(5):614–618. 23584090 10.1038/nm.3174

[61] Li X, Larsson P, Ljuslinder I, Ling A, Löfgren-Burström A, Zingmark C, Edin S, Palmqvist R: A modified protein marker panel to identify four consensus molecular subtypes in colorectal cancer using immunohistochemistry. Pathol Res Pract 2021, 220:153379. 33721619 10.1016/j.prp.2021.153379

[62] Cercek A, Lumish M, Sinopoli J, Weiss J, Shia J, Lamendola-Essel M, El Dika IH, Segal N, Shcherba M, Sugarman R : PD-1 Blockade in Mismatch Repair–Deficient, Locally Advanced Rectal Cancer. N Engl J Med 2022, 386(25):2363–2376. 35660797 10.1056/NEJMoa2201445 PMC9492301

[63] Chalabi M, Fanchi LF, Dijkstra KK, Van den Berg JG, Aalbers AG, Sikorska K, Lopez-Yurda M, Grootscholten C, Beets GL, Snaebjornsson P : Neoadjuvant immunotherapy leads to pathological responses in MMR-proficient and MMR-deficient early-stage colon cancers. Nat Med 2020, 26(4):566–576. 32251400 10.1038/s41591-020-0805-8

[64] de Rosa N, Rodriguez-Bigas MA, Chang GJ, Veerapong J, Borras E, Krishnan S, Bednarski B, Messick CA, Skibber JM, Feig BW : DNA Mismatch Repair Deficiency in Rectal Cancer: Benchmarking Its Impact on Prognosis, Neoadjuvant Response Prediction, and Clinical Cancer Genetics. J Clin Oncol 2016, 34(25):3039–3046. 27432916 10.1200/JCO.2016.66.6826 PMC5012714

[65] Cercek A, Dos Santos Fernandes G, Roxburgh CS, Ganesh K, Ng S, Sanchez-Vega F, Yaeger R, Segal NH, Reidy-Lagunes DL, Varghese AM : Mismatch Repair-Deficient Rectal Cancer and Resistance to Neoadjuvant Chemotherapy. Clin Cancer Res 2020, 26(13):3271–3279. 32144135 10.1158/1078-0432.CCR-19-3728 PMC7348681

[66] Topatana W, Juengpanich S, Li S, Cao J, Hu J, Lee J, Suliyanto K, Ma D, Zhang B, Chen M : Advances in synthetic lethality for cancer therapy: cellular mechanism and clinical translation. J Hematol Oncol 2020, 13(1):118. 32883316 10.1186/s13045-020-00956-5 PMC7470446

[67] Nguyen HT, Duong H-Q: The molecular characteristics of colorectal cancer: Implications for diagnosis and therapy. Oncol Lett 2018, 16(1):9–18. 29928381 10.3892/ol.2018.8679 PMC6006272

